# Update of the global distribution of human gammaherpesvirus 8 genotypes

**DOI:** 10.1038/s41598-021-87038-9

**Published:** 2021-04-07

**Authors:** Amanda de Oliveira Lopes, Natália Spitz, Christian Robson de Souza Reis, Vanessa Salete de Paula

**Affiliations:** 1 Laboratory of Molecular Virology, Oswaldo Cruz Institute, Oswaldo Cruz Foundation, 4365, Brasil Av., Manguinhos, Rio de Janeiro, RJ 21040-360 Brazil; 2 Microbiology Department, Aggeu Magalhães Institute, Oswaldo Cruz Foundation, Pernambuco, 50670-420 Brazil

**Keywords:** Molecular evolution, Phylogenetics, Genotype, Herpes virus, Viral epidemiology, Viral evolution

## Abstract

Human gammaherpesvirus 8 (HHV-8) consists of six major clades (A–F) based on the genetic sequence of the open reading frame (ORF)-K1. There are a few conflicting reports regarding the global distribution of the different HHV-8 genotypes. This study aimed to determine the global distribution of the different HHV-8 genotypes based on phylogenetic analysis of the ORF-K1 coding region using sequences published in the GenBank during 1997–2020 and construct a phylogenetic tree using the maximum likelihood algorithm with the GTR + I + G nucleotide substitution model. A total of 550 sequences from 38 countries/origins were analysed in this study. Genotypes A and C had similar global distributions and were prevalent in Africa and Europe. Genotype B was prevalent in Africa. Of the rare genotypes, genotype D was reported in East Asia and Oceania and genotype E in South America, while genotype F was prevalent in Africa. The highest genotypic diversity was reported in the American continent, with Brazil housing five HHV-8 genotypes (A, B, C, E, and F). In this study, we present update of the global distribution of HHV-8 genotypes, providing a basis for future epidemiological and evolutionary studies of HHV-8.

## Introduction

Human gammaherpesvirus 8 (HHV-8) is known to cause Kaposi’s sarcoma, which is one of the most common cancers in human immunodeficiency virus-infected patients^1^, and the most common form of neoplasia in children from endemic regions, such as the Central, Eastern, and Southern Africa^2^. Kaposi’s sarcoma is responsible for significant morbidity and mortality, with 41,799 new cases and 19,902 deaths reported worldwide in 2018^3^. HHV-8 infection is also associated with other malignancies, including primary effusion lymphoma, multicentric Castleman disease, HHV-8 positive diffuse large B-cell lymphoma, and germinotropic lymphoproliferative disorder. These cancers usually occur in immunodeficient patients, but may also affect immunocompetent individuals^4^.

HHV-8 has a highly conserved double-stranded DNA genome of approximately 140 kilobases; however, both ends of the genome show significant variability. Phylogenetic studies based on the highly variable open reading frame (ORF)-K1 from the 5′ end to the 3′ end led to the identification of six main clades, namely A, B, C, D, E, and F. The ORF-K1 coding region is composed of approximately 870 base pairs (bp) whose sequences differ by up to 30% at the amino acid level^5–7^. This region encodes a transmembrane protein of approximately 289 amino acid residues, which has multiple roles in cellular signal transduction, viral reactivation, endothelial cell immortalisation, and host immune recognition^7,8^.

Studies have reported that the different genotypes have variable penetrance in different human populations of distinct ethnic and geographic groups^5,9^. However, there are only a few studies that have reported the geographic distribution of the different HHV-8 genotypes, these studies are old with outdated information, and phylogenetic analyses were performed with a smaller number of sequences. In this study, we aimed to determine the worldwide distribution of different HHV-8 genotypes based on phylogenetic analysis of the ORF-K1 coding region using a greater number of sequences published in GenBank. This study is an update and expansion of previous efforts to generate a global distribution of HHV-8 genotypes based on the ORF-K1 coding region.

## Methods

Previous publications that have reported different HHV-8 genotypes based on the sequence of the ORF-K1 coding region and sequences of the ORF-K1 coding region deposited in the GenBank (http://www.ncbi.nlm.nih.gov/) were used to obtain a database of sequences of the different HHV-8 genotypes (A to F). A literature search was performed using the search terms: HHV-8 genotype ORF-K1, HHV-8 genotyping ORF-K1, HHV-8 molecular epidemiology, and HHV-8 genetic diversity. The sequences were found in the GenBank using the search words: HHV-8, ORF-K1, K1, and gene. All sequences published from 1997 to August 2020 were included to build the database. ORF-K1 sequences smaller than 730 bp and sequences without information about country or origin were excluded from the analysis.

All sequences from this study were aligned using MUSCLE software^10^ included in the MEGA program (version 7)^11^. The origin of each sequence was designated according to the locality from where the sample was collected. HHV-8 genotypes reported in a previous study or submitted to GenBank were taken as is.

A phylogenetic tree was inferred using the online version of the PhyML program^12^ using the maximum likelihood method under the GTR + I + G nucleotide substitution model selected by the Smart Model Selection in PhyML^13^. A heuristic tree search was performed using the subtree pruning-re-grafting branch-swapping algorithm, and the reliability of the phylogenies was estimated with aLRT^14^ based on a Shimodaira-Hasegawa-like procedure (SH-aLRT). We used iToL to view phylogenetic tree and GraphPad Prism 8 to compute and graph the diversity of HHV-8 genotypes present in each country/origin.

## Results

**Figure 1:**
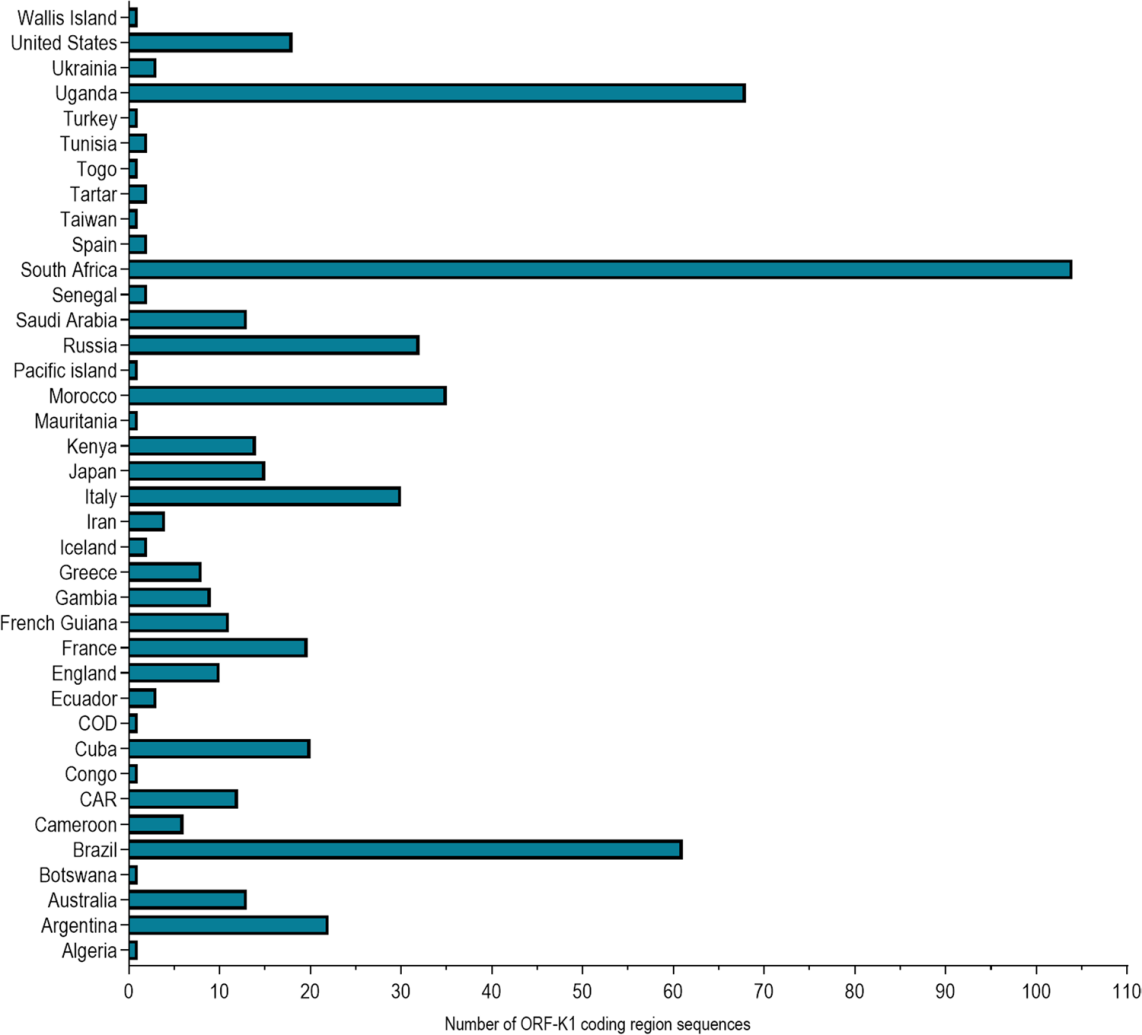
Number of HHV-8 ORF-K1 coding region sequences published per country/origin. Sequences were collected from GenBank. (CAR, Central African Republic; COD, Democratic Republic of the Congo).

Based on a literature review using PubMed, we identified 30 studies from 1997 to 2020 that had published sequences of the ORF-K1 coding region. These sequences deposited in GenBank from these studies were combined with other sequences from this database, resulting in a total of 550 sequences from 38 countries/origins (Supplementary material 1). Figure 1 shows the number of sequences of the HHV-8 ORF-K1 coding region published per country/origin.

**Figure 2:**
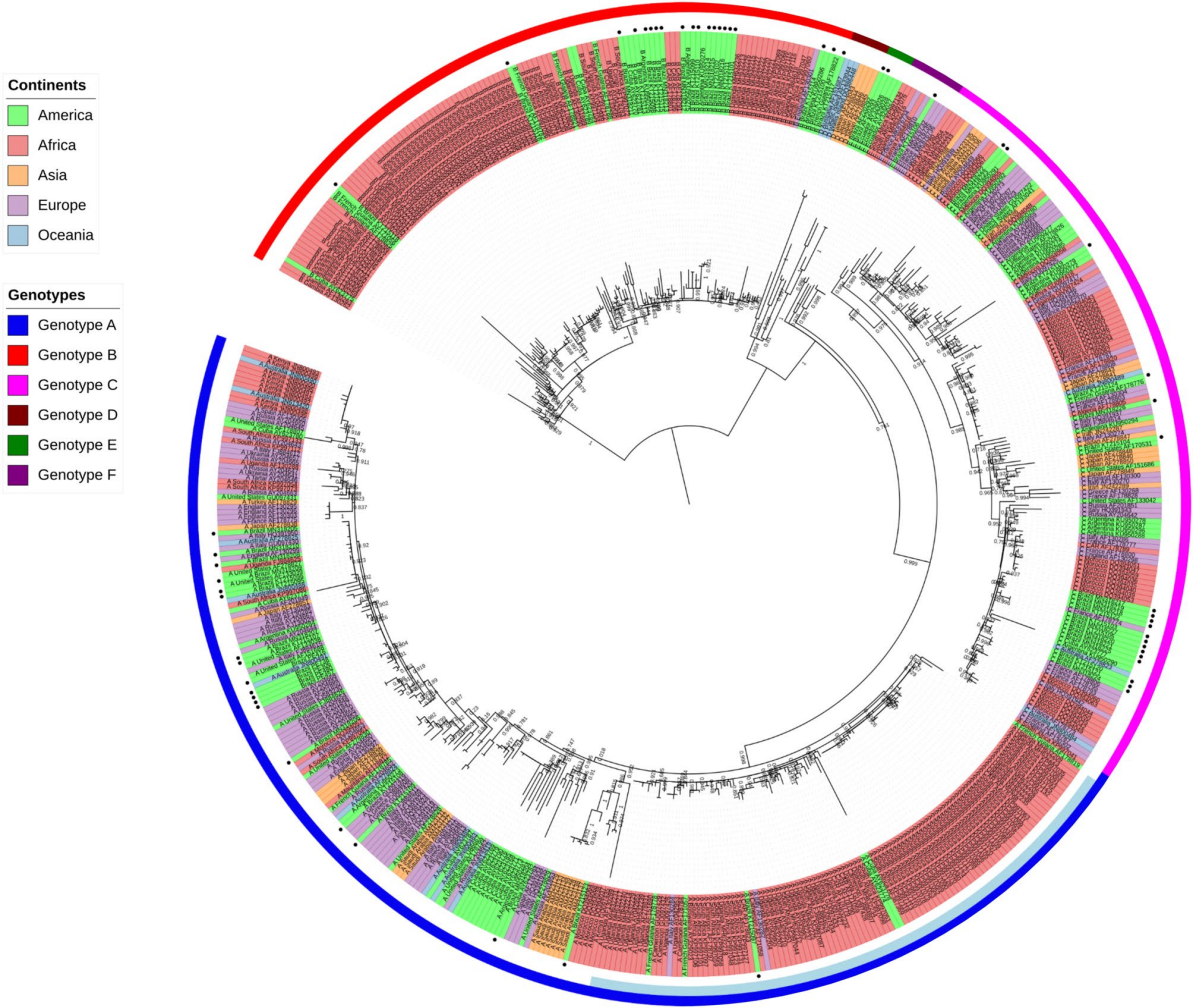
Phylogenetic analysis of multiple HHV-8 sequences based on the sequence of ORF-K1 coding region using the maximum-likelihood method. GenBank accession numbers for all sequences are presented in the supplementary material 1. The colours indicating the different genotypes and continents are shown in the key for the figure. The colour light blue refers to the subgenotype A5. The sequences are indicated by the genotype followed by country or origin (CAR, Central African Republic; COD, Democratic Republic of the Congo), and accession number. The filled circle symbol indicates location of all Brazilian sequences. In this phylogenetic tree are indicated all the values at internal nodes likelihood-ratio test value for the branch. The tree is rooted in the midpoint and was viewed using iToL.

We performed a phylogenetic reconstruction using the 550 sequences to confirm the presence of the six known HHV-8 genotypes and estimate the evolutionary relationships between them. Figure 2 (and Supplementary material 2) shows the HHV-8 phylogenetic tree constructed using the ORF-K1 coding region sequences. The phylogenetic analysis clearly distinguished the six known clades (A, B, C, D, E, and F), and all the branches had high approximate likelihood-ratio test (aLRT) values.

As shown in Table 1, among the 550 sequences, genotype A showed the highest prevalence (262, 47.6%), followed by genotype C (141, 25.6%), genotype B (125, 22.7%), genotype F (10, 1.8%), genotype D (7, 1.3%), and genotype E (5, 0.9%). Most of the sequences had originated in Africa (258; 46.9%), followed by America (135; 12.4%), Europe (108; 19.6%), Asia (34; 6.2%), and Oceania (15; 2.7%). In the Table 1, the data in bold indicates the distribution of HHV-8 genotypes by continent and globe.

**Table 1:** Genotypic distribution of HHV-8 by geographic regions.

Origin	Sequences		Genotypes											
			A		B		C		D		E		F	
	n	%	n	%	n	%	n	%	n	%	n	%	n	%
***Africa***	**258**	**46.9**	**124**	**48.1**	**89**	**34.5**	**41**	**15.9**	**0**	**0**	**0**	**0**	**4**	**1.6**
Algeria	1	0.2	0	0	0	0	1	100	0	0	0	0	0	0
Botswana	1	0.2	1	100	0	0	0	0	0	0	0	0	0	0
Cameroon	6	1.1	6	100	0	0	0	0	0	0	0	0	0	0
Central African Republic	12	2.2	5	41.7	5	41.7	2	16.7	0	0	0	0	0	0
Congo	1	0.2	0	0	1	100	0	0	0	0	0	0	0	0
Democratic Republic of the Congo	1	0.2	0	0	1	100	0	0	0	0	0	0	0	0
Gambia	9	1.6	0	0	9	100	0	0	0	0	0	0	0	0
Kenya	14	2.5	12	85.7	0	0	1	7.1	0	0	0	0	1	7.1
Mauritania	1	0.2	1	100	0	0	0	0	0	0	0	0	0	0
Morocco	35	6.4	5	14.3	0	0	30	85.7	0	0	0	0	0	0
Senegal	2	0.4	0	0	1	50	1	50	0	0	0	0	0	0
South Africa	104	18.9	58	55.8	44	42.3	0	0	0	0	0	0	2	1.9
Togo	1	0.2	0	0	1	100	0	0	0	0	0	0	0	0
Tunisia	2	0.4	0	0	0	0	2	100	0	0	0	0	0	0
Uganda	68	12.4	36	52.9	27	39.7	4	5.9	0	0	0	0	1	1.5
***America***	**135**	**12.4**	**52**	**38.5**	**35**	**25.9**	**42**	**31.1**	**0**	**0**	**5**	**3.7**	**1**	**0.7**
Argentina	22	4.0	3	13.6	4	18.2	15	68.2	0	0	0	0	0	0
Brazil	61	11.1	19	31.1	20	32.8	19	31.1	0	0	2	3.3	1	1.6
Cuba	20	3.6	14	70	5	25.0	1	5.0	0	0	0	0	0	0
Ecuador	3	0.5	0	0	0	0	0	0	0	0	3	100	0	0
French Guiana	11	2.0	4	36.4	6	54.5	1	9.1	0	0	0	0	0	0
United States	18	3.3	12	66.7	0	0	6	33.3	0	0	0	0	0	0
***Asia***	**34**	**6**.**2**	**16**	**47**.**1**	**0**	**0**	**14**	**41**.**2**	**4**	**11**.**8**	**0**	**0**	**0**	**0**
Iran	4	0.7	0	0	0	0	4	100	0	0	0	0	0	0
Japan	15	2.7	5	33.3	0	0	7	46.7	3	20	0	0	0	0
Saudi Arabia	13	2.4	10	76.9	0	0	3	23.1	0	0	0	0	0	0
Taiwan	1	0.2	0	0	0	0	0	0	1	100	0	0	0	0
Turkey	1	0.2	1	100	0	0	0	0	0	0	0	0	0	0
***Europe***	**108**	**19.6**	**61**	**56.5**	**1**	**0.9**	**41**	**38.0**	**0**	**0**	**0**	**0**	**5**	**4.6**
England	10	1.8	5	50	1	10	4	40	0	0	0	0	0	0
France	19	3.5	1	5.3	0	0	13	68.4	0	0	0	0	5	26.3
Greece	8	1.5	4	50	0	0	4	50	0	0	0	0	0	0
Iceland	2	0.4	1	50	0	0	1	50	0	0	0	0	0	0
Italy	30	5.5	18	60	0	0	12	40	0	0	0	0	0	0
Russia	32	5.8	26	81.3	0	0	6	18.8	0	0	0	0	0	0
Spain	2	0.4	2	100	0	0	0	0	0	0	0	0	0	0
Tartar	2	0.4	2	100	0	0	0	0	0	0	0	0	0	0
Ukraine	3	0.5	2	66.7	0	0	1	33.3	0	0	0	0	0	0
***Oceania***	**15**	**2**.**7**	**9**	**60**	**0**	**0**	**3**	**20**	**3**	**20**	**0**	**0**	**0**	**0**
Australia	13	2.4	9	69.2	0	0	3	23.1	1	7.7	0	0	0	0
Pacific island	1	0.2	0	0	0	0	0	0	1	100	0	0	0	0
Wallis Island	1	0.2	0	0	0	0	0	0	1	100	0	0	0	0
***Global***	**550**	**100**	**262**	**47.6**	**125**	**22.7**	**141**	**25.6**	**7**	**1.3**	**5**	**0.9**	**10**	**1.8**

**Figure 3:**
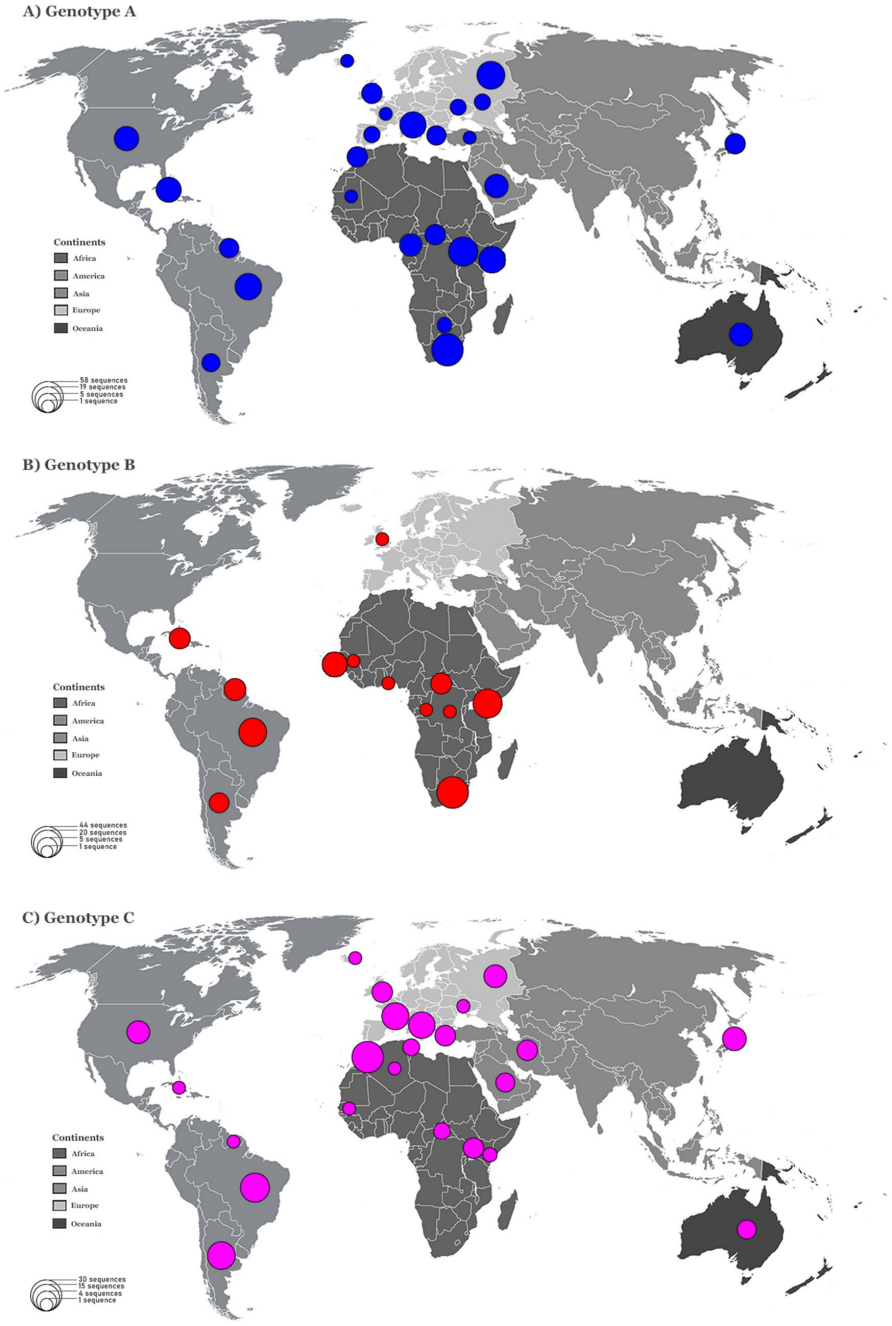

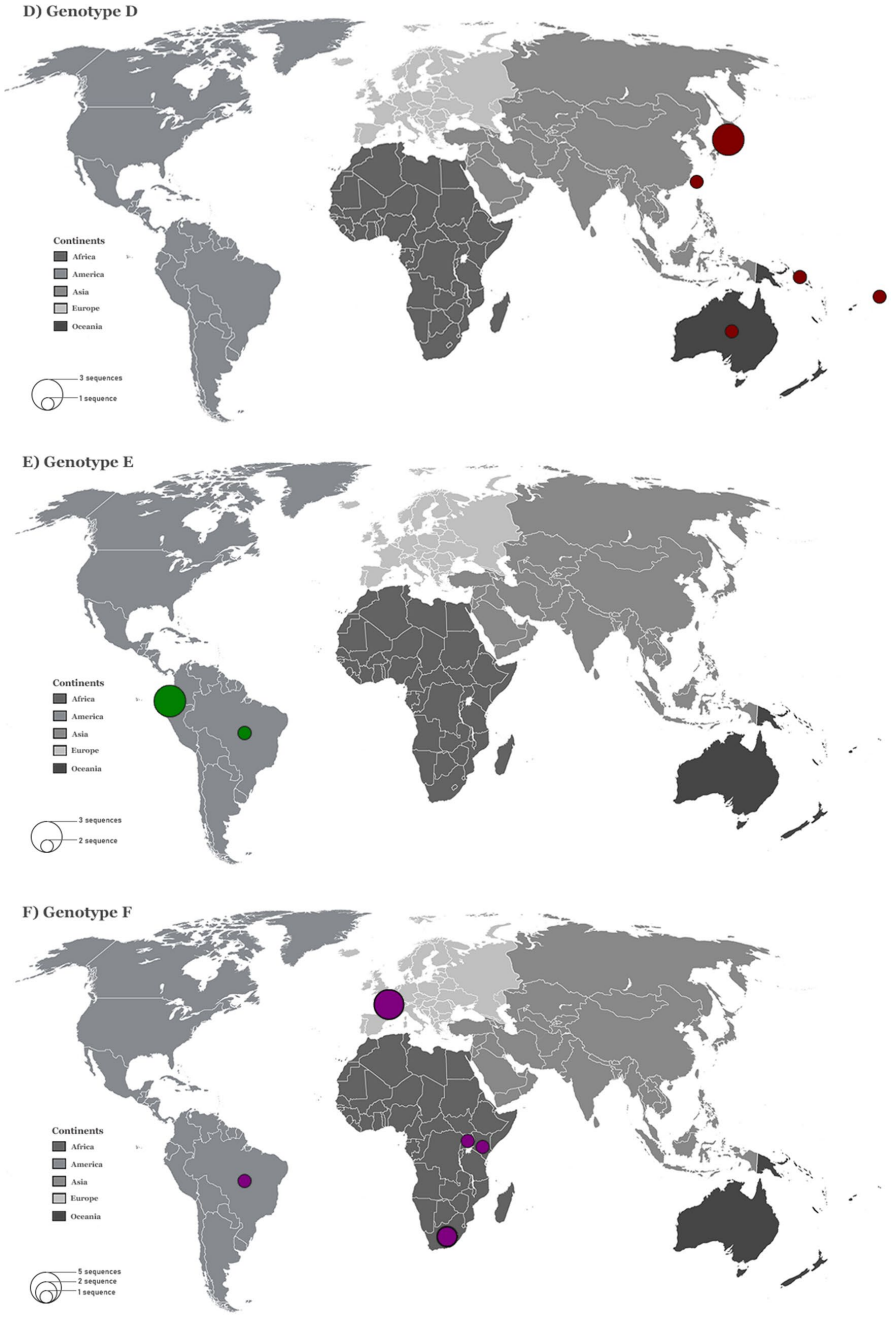
Global distribution of HHV-8 genotypes. The six world maps demonstrate the distribution of each HHV-8 genotypes (**A** - **F**). The genotypes and continents are indicated in the figure. Countries forming a continent are shaded in the same colour. The size of the circles is proportional to the number of HHV-8 ORF-K1 coding region sequences belonging to a particular origin analysed in this study. All sequences collected from GenBank.

HHV-8 sequences were found in all continents except Antarctica (Table 1). Genotypes A and C were identified in all continents and showed a similar global distribution. Both genotypes were prevalent in Africa and Europe, followed by America, Asia, and Oceania (Fig. 3A,C). The subgenotype A5 was responsible by the greater prevalence of genotype A in the African continent, and was also found in Europe and America. Genotypes B and F were prevalent in Africa, but were also identified in America and Europe (Fig. 3B,F). Genotype D was only reported in East Asia and Oceania, while genotype E was reported only in America (Fig. 3D,E).

**Figure 4:**
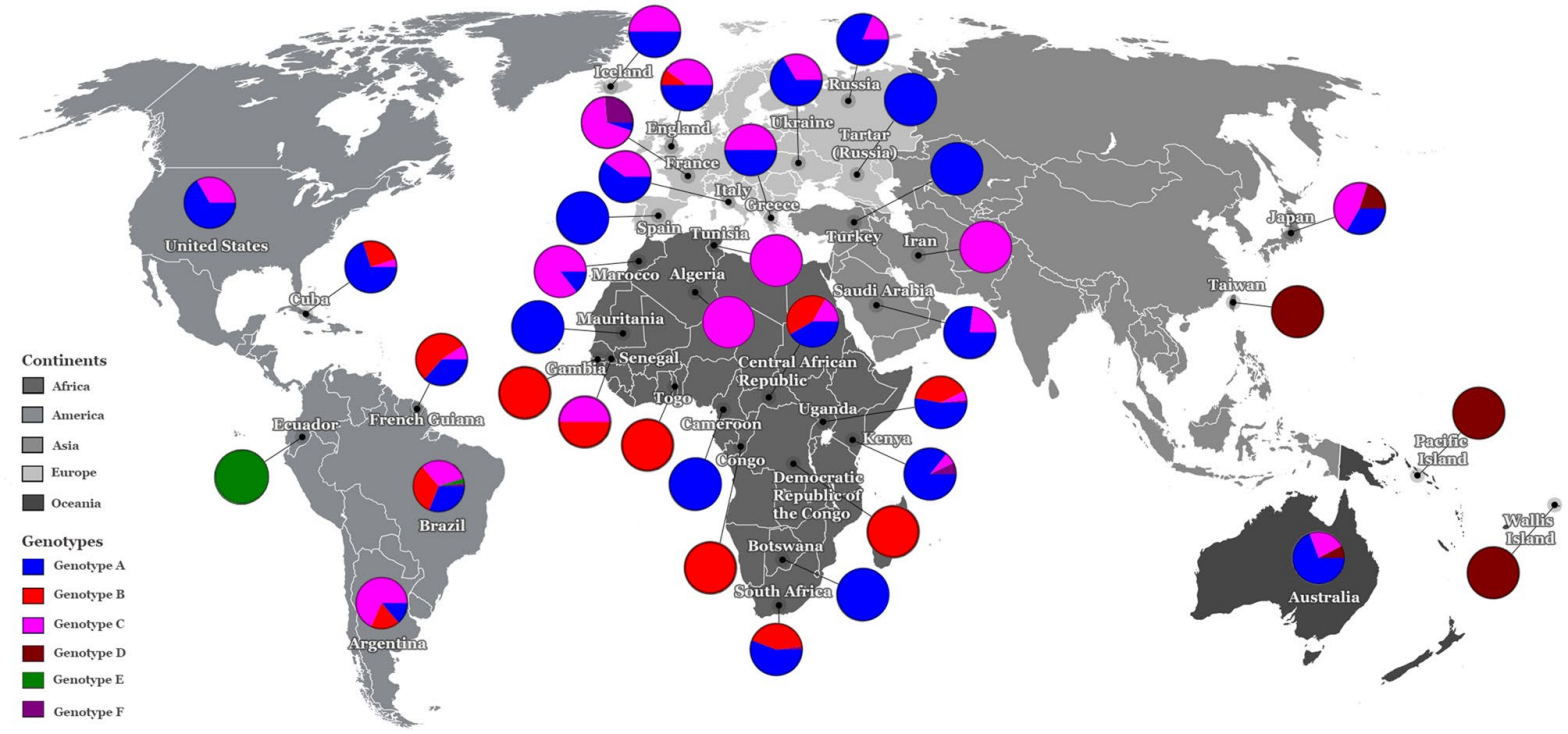
The HHV-8 genotypic diversity per country/origin. The pie charts indicate the proportion of occurrence of the different HHV-8 genotypes in the respective countries/origins. The genotypes and continents are indicated in the figure. Countries from the same region are shaded in the same colour on the world map. These distributions of HHV-8 genotypes were based on sequences analysed in this study.

The American continent exhibited the highest genotypic diversity with five genotypes (A, B, C, E, and F) (Fig. 4), wherein the majority of the American countries, including Argentina, Brazil, Cuba, and French Guiana, had at least three genotypes. Such a high degree of diversity was not observed in the other continents (Fig. 4).

In the American continent, the highest HHV-8 genotypic diversity was found in Brazil, wherein five of the six major genotypes were identified (Fig. 4). Our phylogenetic analysis contained 61 sequences that originated from Brazil, which is the third country with the largest number of published ORF-K1 coding region sequences, behind only Uganda (68 sequences) and South Africa (104 sequences), both of which are African countries.

## Discussion

Till date, this is the largest study to determine the global distribution of HHV-8 genotypes. A phylogenetic analysis was performed on a total of 550 distinct sequences from 38 countries/origins, indicating the large global coverage of our study.

From our phylogenetic reconstruction (Fig. 2), we obtained new insights on the global distribution of HHV-8 genotypes. First, genotypes A and C showed similar global distributions and were highly prevalent in Europe and Africa. Genotype B was found to be prevalent in Africa and was also identified in Central and South America and Europe. Of the rarer genotypes, genotype D was reported in East Asia and Oceania and genotype E was reported in Amerindian populations of South America. Moreover, the highly uncommon genotype F was identified in Africa, America, and Europe (Fig. 3).

Previous studies have shown that the distribution of different HHV-8 ORF-K1 genotypes between geographic and ethnic backgrounds appears to depend on the global spread of modern human populations^15,16^. Based on recent studies of contemporary human migrations^17–19^, and the HHV-8 phylogenetic reconstruction shown in Fig. 2, we found that genotype B is the most ancient genotype with four major branches diverging from it (Fig. 2). Previous reports have stated that genotype B may have originated with the first migration of modern humans in Africa^15,16^. We hypothesize that HHV-8 started to spread and evolve together with their human hosts as they migrated towards Northeast and Southwest Africa approximately 130–110 thousand years (kilo annum, ka) ago due to regional climate changes^17^. Subsequently, the several migrations that have occurred in Africa over thousands of years^17^ may also explain the predominance of genotype B in this continent (Fig. 3B). The slave trade, which occurred between the sixteenth and nineteenth century, due to colonisation of the American continent by Europeans^20^ supports the identification of this genotype in America (Fig. 3B).

After human expansion out of Africa, genotype D, which is evolutionarily the closest to genotype B (Fig. 2), probably evolved in isolated populations of the first human migrants in East Asia approximately 40 ka ago, in Australasia approximately 40 ka ago^17–19^, and in Melanesia Islands approximately 3.4 ka ago^21^, explaining the dispersion of this genotype in the globe (Fig. 3d). Genotype E is evolutionarily closest to genotype D (Fig. 2) and was identified only in specific groups of South American Amerindians isolated from East Ecuador and Northern Brazil (Fig. 3E)^22,23^. Thus, genotype E possibly originated in human populations that had migrated across the Bering Strait from Asia approximately 20–15 ka ago^18^. Furthermore, the evolutionarily ancient genotype F, which may have diverged after genotype D (Fig. 2), was localised in the Bantu Gisu tribe in eastern Uganda, as shown in a previous study^24^. Therefore, genotype F may also have had an African genesis after the dispersal of modern humans, which occurred more than 40 ka ago in this continent^17,19^. In this study, we did not find a large global distribution of genotypes D, E, and F; these genotypes probably remained in isolated populations of different ethnic groups (Fig. 3F)^22–24^.

Genotypes A and C have a common ancestor with genotype F and may have diverged after genotype E (Fig. 2). Some older sequences of genotypes A and C were found to have localised in Africa (Fig. 2) and, thus the genotypes may have diverged more recently in this continent after a greater spread of contemporary human populations^17^ approximately 20 to 15 ka ago^18^, and this is different from what is indicated by previous studies^15,16,24,25^. This novel finding is based on a high number of HHV-8 genotypic sequences analysed in this study (Fig. 1) in association with recent studies of modern human migrations^17–19^. In addition, the dispersion of genotypes A and C possibly occurred in Africa for thousands of years due to several migrations that had occurred in this continent^17^, arriving in the Middle East (Western Asia) and Europe in waves of migration 8 ka ago^26^. This may explain why these genotypes are prevalent in African and European countries, and identified in regions subsequently occupied or colonised by these countries, such as American countries (in the late 1400 s)^27^ and Australia (in the late 1700s)^20^. The waves of human migration due to the European colonisation of the world also justify the distribution of these genotypes on five continents (Fig. 3A,C)^27^.

Furthermore, in this study, a high HHV-8 genotypic diversity was observed in the American countries/origin, wherein Brazil with five HHV-8 genotypes (A, B, C, E, and F) had the highest genotypic diversity of the 38 countries/origins analysed in this study (Fig. 4). This high diversity is possibly a result of human migrations started at least 15 ka ago, with genotype E probably arriving first in this continent^18^. Thereafter, extensive migrations since the colonial era due to slave trade after the late 1400s and in the last two centuries may have contributed to the spread of genotypes A, B, C, and F in America. In fact, 5 million people from more than 60 countries moved to Brazil at the end of the 1800s and in the beginning of the 1900s^7,27^. These conclusions are in agreement with a previous study that analysed genetic distances and showed that genotypes A, B, and C were possibly brought into Brazil by immigrants from several countries, including Africa, Europe, Asia, and Oceania^7^. Nevertheless, as many HHV-8 sequences were obtained from HIV infected individuals, some routes of HHV-8 transmission and spread globally may have been more recent through sexual contact on a trip or sex with visiting tourists^7^. This high HHV-8 diversity found in Brazil may also be a result of high number of published ORF-K1 coding region sequences from this country (total of 61 sequences) (Fig. 1 and Table 1). Further studies with additional HHV-8 sequences from other regions and countries, including phylogeographic analysis, can prove to be very informative to understand the HHV-8 route of transmission and diversity in the globe.

The subgenotype A5 was also analysed in this study once this subgenotype was described to be common in Africa. We confirmed this information with our phylogenetic analysis (Fig. 2), where subgenotype A5 was responsible by the greater prevalence of genotype A in the African continent. The clade A5 was the most ancient genotype A (Fig. 2) and may have diverged in other subgenotypes after greater spread of contemporary human populations^17^ to the Middle East (Western Asia) and Europe in 8 ka ago^26^.

The World Health Organisation has reported new cancers that are associated with HHV-8 infections^4^. Unfortunately, in our study, we were unable to perform a correlation analysis between genotypes and tumorigenic potential, due to presence of only a few studies with conflicting data^7^. However, we believe that this interaction should be investigated in future studies, as the transmembrane K1 glycoprotein encoded by the viral ORF-K1 gene can contribute to the pathogenesis of HHV-8-associated human cancers^28^.

In conclusion, we analysed 550 ORF-K1 sequences and revealed the current global distribution of different HHV-8 genotypes. The new insights about the circulation and molecular evolution of HHV-8 genotypes are: the genotypes A and C were prevalent in Africa and Europe, had probably originated in Africa, and may have diverged more recently in this continent; while the rare genotype F was prevalent in Africa which may have diverged after genotype D; and the Brazil was found to be the country with the highest HHV-8 genotypic diversity in the world. Our study provides a basis for future studies investigating the molecular epidemiology and genetic evolution of HHV-8.

## Supplementary Information

Supplementary Information 1.

Supplementary Information 2.

## Data Availability

The datasets used to support the findings of this study are available from the corresponding author upon request.
