# Supplementary material for: Update of the global distribution of human gammaherpesvirus 8 genotypes
Source: Sci Rep. 2021 Apr 7;11:7640. doi: 10.1038/s41598-021-87038-9 (PMC8026617; doi:10.1038/s41598-021-87038-9)
Supplement: Supplementary file 1 — Supplementary Information 1. [file 41598_2021_87038_MOESM1_ESM.pdf]

Update of the global distribution of Human gammaherpesvirus 8 genotypes

Amanda de Oliveira Lopes, Natália Spitz, Christian Robson de Souza Reis, Vanessa Salete de Paula

Supplementary material 1. GenBank accession numbers and references from 550 ORF-K1 coding region sequences regarding HHV-8 genotypes (A to F) and country/origin.

Genotype	GenBank accession number	Country	Reference*
A	KU950273	Argentina	(Pérez and Tous, 2017)
A	KU950281	Argentina	(Pérez and Tous, 2017)
A	KU950284	Argentina	(Pérez and Tous, 2017)
A	AF278824	Australia	(Meng et al., 1999)
A	AF278825	Australia	(Meng et al., 1999)
A	JN613419	Australia	(Speicher et al., 2013)
A	JN613420	Australia	(Speicher et al., 2013)
A	JN800483	Australia	Unpublished
A	JN800485	Australia	Unpublished
A	JN800488	Australia	Unpublished
A	JN800490	Australia	Unpublished
A	JN800491	Australia	Unpublished
A	AY329023	Botswana	(Whitby et al., 2004)
A	KT215095	Brazil	(Tozetto-Mendoza et al., 2016)
A	KT215096	Brazil	(Tozetto-Mendoza et al., 2016)
A	KT215097	Brazil	(Tozetto-Mendoza et al., 2016)
A	KT215107	Brazil	(Tozetto-Mendoza et al., 2016)
A	KT215108	Brazil	(Tozetto-Mendoza et al., 2016)
A	KT215109	Brazil	(Tozetto-Mendoza et al., 2016)
A	KT215123	Brazil	(Tozetto-Mendoza et al., 2016)
A	MN318207	Brazil	(Lopes et al., 2020)
A	MN318208	Brazil	(Lopes et al., 2020)
A	MN318209	Brazil	(Lopes et al., 2020)
A	MN318206	Brazil	(Lopes et al., 2020)
A	MN318210	Brazil	(Lopes et al., 2020)
A	MN318205	Brazil	(Lopes et al., 2020)
A	MN318204	Brazil	(Lopes et al., 2020)
A	MT597155	Brazil	(in preparation)
A	MT597156	Brazil	(in preparation)
A	MT597157	Brazil	(in preparation)
A	MT597158	Brazil	(in preparation)
A	MT597159	Brazil	(in preparation)
A	AF171058	Cameroon	(Fouchard et al., 2000)
A	AF178784	Cameroon	(Fouchard et al., 2000)

A	AF178797	Cameroon	(Fouchard et al., 2000)
A	FJ884607	Cameroon	(Tornesello et al., 2010)
A	GU097429	Cameroon	(Tornesello et al., 2010)
A	GU097432	Cameroon	(Tornesello et al., 2010)
A	AF171059	Central African Republic	(Fouchard et al., 2000)
A	AF178779	Central African Republic	(Lacoste et al., 2000a)
A	AF178790	Central African Republic	(Lacoste et al., 2000a)
A	AF178798	Central African Republic	(Lacoste et al., 2000a)
A	AF178823	Central African Republic	(Lacoste et al., 2000a)
A	AY623768	Cuba	Unpublished
A	AY623769	Cuba	Unpublished
A	AY623771	Cuba	Unpublished
A	AY623772	Cuba	Unpublished
A	AY623773	Cuba	Unpublished
A	AY623774	Cuba	Unpublished
A	AY623776	Cuba	Unpublished
A	AY623778	Cuba	Unpublished
A	AY623779	Cuba	Unpublished
A	AY623780	Cuba	Unpublished
A	AY623782	Cuba	Unpublished
A	AY623785	Cuba	Unpublished
A	AY623787	Cuba	Unpublished
A	AY623790	Cuba	Unpublished
A	AF130295	England	(Cook et al., 1999)
A	AF130296	England	(Cook et al., 1999)
A	AF130297	England	(Cook et al., 1999)
A	AF130299	England	(Cook et al., 1999)
A	AF130303	England	(Cook et al., 1999)
A	AF178775	France	(Lacoste et al., 2000a)
A	AF178778	French Guiana	(Lacoste et al., 2000a)
A	AF178785	French Guiana	(Lacoste et al., 2000a)
A	AF178819	French Guiana	(Lacoste et al., 2000a)
A	AF171057	French Guiana	(Fouchard et al., 2000)
A	FJ884608	Greece	(Tornesello et al., 2010)
A	FJ884610	Greece	(Tornesello et al., 2010)
A	GU097419	Greece	(Tornesello et al., 2010)
A	GU097420	Greece	(Tornesello et al., 2010)
A	AF130272	Iceland	(Cook et al., 1999)
A	AF130269	Italy	(Cook et al., 1999)
A	AF130282	Italy	(Cook et al., 1999)
A	AF130283	Italy	(Cook et al., 1999)
A	AF130284	Italy	(Cook et al., 1999)
A	AF130285	Italy	(Cook et al., 1999)
A	FJ884611	Italy	(Tornesello et al., 2010)

A	FJ884612	Italy	(Tornesello et al., 2010)
A	FJ884613	Italy	(Tornesello et al., 2010)
A	FJ884615	Italy	(Tornesello et al., 2010)
A	GU097421	Italy	(Tornesello et al., 2010)
A	GU097430	Italy	(Tornesello et al., 2010)
A	HQ391919	Italy	(Mancuso et al., 2011)
A	HQ391920	Italy	(Mancuso et al., 2011)
A	HQ391923	Italy	(Mancuso et al., 2011)
A	HQ391924	Italy	(Mancuso et al., 2011)
A	HQ391934	Italy	(Mancuso et al., 2011)
A	HQ391935	Italy	(Mancuso et al., 2011)
A	HQ391950	Italy	(Mancuso et al., 2011)
A	AF178786	Japan	(Lacoste et al., 2000a)
A	AF278838	Japan	(Meng et al., 2001)
A	AF278839	Japan	(Meng et al., 2001)
A	AF278851	Japan	(Meng et al., 2001)
A	AF278852	Japan	(Meng et al., 2001)
A	GU097427	Kenya	(Tornesello et al., 2010)
A	GU097433	Kenya	(Tornesello et al., 2010)
A	GU097435	Kenya	(Tornesello et al., 2010)
A	JN800492	Kenya	Unpublished
A	JN800493	Kenya	Unpublished
A	JN800494	Kenya	Unpublished
A	JN800495	Kenya	Unpublished
A	JN800496	Kenya	Unpublished
A	JN800497	Kenya	Unpublished
A	JN800498	Kenya	Unpublished
A	JN800499	Kenya	Unpublished
A	JN800500	Kenya	Unpublished
A	AF178812	Mauritania	(Lacoste et al., 2000a)
A	DQ394037	Marocco	(Duprez et al., 2006b)
A	DQ394038	Marocco	(Duprez et al., 2006b)
A	DQ394057	Marocco	(Duprez et al., 2006b)
A	DQ394064	Marocco	(Duprez et al., 2006b)
A	DQ394068	Marocco	(Duprez et al., 2006b)
A	AF201847	Russia	(Lacoste et al., 2000b)
A	AF201848	Russia	(Lacoste et al., 2000b)
A	AF201849	Russia	(Lacoste et al., 2000b)
A	AF201850	Russia	(Lacoste et al., 2000b)
A	AF201852	Russia	(Lacoste et al., 2000b)
A	AF201853	Russia	(Lacoste et al., 2000b)
A	AY204643	Russia	(Kadyrova et al., 2003)
A	AY204644	Russia	(Kadyrova et al., 2003)
A	AY204645	Russia	(Kadyrova et al., 2003)
A	AY204647	Russia	(Kadyrova et al., 2003)
A	AY204648	Russia	(Kadyrova et al., 2003)
A	AY204650	Russia	(Kadyrova et al., 2003)
A	AY204652	Russia	(Kadyrova et al., 2003)
A	AY204653	Russia	(Kadyrova et al., 2003)

A	AY204654	Russia	(Kadyrova et al., 2003)
A	AY204655	Russia	(Kadyrova et al., 2003)
A	AY204660	Russia	(Kadyrova et al., 2003)
A	AY204662	Russia	(Kadyrova et al., 2003)
A	AY204663	Russia	(Kadyrova et al., 2003)
A	AY204664	Russia	(Kadyrova et al., 2003)
A	AY204665	Russia	(Kadyrova et al., 2003)
A	AY204667	Russia	(Kadyrova et al., 2003)
A	AY204668	Russia	(Kadyrova et al., 2003)
A	AY204670	Russia	(Kadyrova et al., 2003)
A	KJ130029	Russia	(Shirokov et al., 2007)
A	KJ583230	Russia	Unpublished
A	AM423127	Saudi Arabia	(Al-Otaibi et al., 2007)
A	AM423128	Saudi Arabia	(Al-Otaibi et al., 2007)
A	AM423129	Saudi Arabia	(Al-Otaibi et al., 2007)
A	AM423131	Saudi Arabia	(Al-Otaibi et al., 2007)
A	AM423132	Saudi Arabia	(Al-Otaibi et al., 2007)
A	AM423133	Saudi Arabia	(Al-Otaibi et al., 2007)
A	AM423134	Saudi Arabia	(Al-Otaibi et al., 2007)
A	AM423137	Saudi Arabia	(Al-Otaibi et al., 2007)
A	AM423138	Saudi Arabia	(Al-Otaibi et al., 2007)
A	AM423139	Saudi Arabia	(Al-Otaibi et al., 2007)
A	AF387369	South Africa	(Treurnicht et al., 2002)
A	KP997035	South Africa	(Isaacs et al., 2016)
A	KP997040	South Africa	(Isaacs et al., 2016)
A	KP997041	South Africa	(Isaacs et al., 2016)
A	KP997044	South Africa	(Isaacs et al., 2016)
A	KP997046	South Africa	(Isaacs et al., 2016)
A	KP997048	South Africa	(Isaacs et al., 2016)
A	KP997049	South Africa	(Isaacs et al., 2016)
A	KP997051	South Africa	(Isaacs et al., 2016)
A	KP997052	South Africa	(Isaacs et al., 2016)
A	KP997053	South Africa	(Isaacs et al., 2016)
A	KP997054	South Africa	(Isaacs et al., 2016)
A	KP997057	South Africa	(Isaacs et al., 2016)
A	KP997058	South Africa	(Isaacs et al., 2016)
A	KP997059	South Africa	(Isaacs et al., 2016)
A	KP997061	South Africa	(Isaacs et al., 2016)
A	KP997063	South Africa	(Isaacs et al., 2016)
A	KP997065	South Africa	(Isaacs et al., 2016)
A	KP997070	South Africa	(Isaacs et al., 2016)
A	KP997073	South Africa	(Isaacs et al., 2016)
A	KP997075	South Africa	(Isaacs et al., 2016)
A	KP997078	South Africa	(Isaacs et al., 2016)
A	KP997083	South Africa	(Isaacs et al., 2016)
A	KP997085	South Africa	(Isaacs et al., 2016)
A	KP997086	South Africa	(Isaacs et al., 2016)
A	KP997087	South Africa	(Isaacs et al., 2016)
A	KP997088	South Africa	(Isaacs et al., 2016)

A	KP997089	South Africa	(Isaacs et al., 2016)
A	KP997090	South Africa	(Isaacs et al., 2016)
A	KP997091	South Africa	(Isaacs et al., 2016)
A	KP997092	South Africa	(Isaacs et al., 2016)
A	KP997093	South Africa	(Isaacs et al., 2016)
A	KP997097	South Africa	(Isaacs et al., 2016)
A	KP997098	South Africa	(Isaacs et al., 2016)
A	KP997099	South Africa	(Isaacs et al., 2016)
A	KP997101	South Africa	(Isaacs et al., 2016)
A	KP997103	South Africa	(Isaacs et al., 2016)
A	KP997105	South Africa	(Isaacs et al., 2016)
A	KP997106	South Africa	(Isaacs et al., 2016)
A	KP997107	South Africa	(Isaacs et al., 2016)
A	KP997109	South Africa	(Isaacs et al., 2016)
A	KP997110	South Africa	(Isaacs et al., 2016)
A	KP997111	South Africa	(Isaacs et al., 2016)
A	KP997112	South Africa	(Isaacs et al., 2016)
A	KP997113	South Africa	(Isaacs et al., 2016)
A	KP997117	South Africa	(Isaacs et al., 2016)
A	KP997118	South Africa	(Isaacs et al., 2016)
A	KP997120	South Africa	(Isaacs et al., 2016)
A	KP997121	South Africa	(Isaacs et al., 2016)
A	KP997123	South Africa	(Isaacs et al., 2016)
A	KP997124	South Africa	(Isaacs et al., 2016)
A	KP997125	South Africa	(Isaacs et al., 2016)
A	KP997126	South Africa	(Isaacs et al., 2016)
A	KP997127	South Africa	(Isaacs et al., 2016)
A	KP997131	South Africa	(Isaacs et al., 2016)
A	KP997132	South Africa	(Isaacs et al., 2016)
A	KP997133	South Africa	(Isaacs et al., 2016)
A	KP997135	South Africa	(Isaacs et al., 2016)
A	AF130305	spain	(Cook et al., 1999)
A	AF178794	spain	(Lacoste et al., 2000a)
A	AY204646	Tartar	(Kadyrova et al., 2003)
A	AY204651	Tartar	(Kadyrova et al., 2003)
A	AF178829	Turkey	(Lacoste et al., 2000a)
A	AF130288	Uganda	(Cook et al., 1999)
A	AF130289	Uganda	(Cook et al., 1999)
A	AF151690	Uganda	(Meng et al., 1999)
A	AF278831	Uganda	(Meng et al., 1999)
A	AY042950	Uganda	(Kakoola et al., 2001)
A	AY042951	Uganda	(Kakoola et al., 2001)
A	AY042952	Uganda	(Kakoola et al., 2001)
A	AY042953	Uganda	(Kakoola et al., 2001)
A	AY042955	Uganda	(Kakoola et al., 2001)
A	FJ884623	Uganda	(Tornesello et al., 2010)
A	FJ884624	Uganda	(Tornesello et al., 2010)
A	FJ884625	Uganda	(Tornesello et al., 2010)
A	GU097426	Uganda	(Tornesello et al., 2010)

A	GU097428	Uganda	(Tornesello et al., 2010)
A	GU097434	Uganda	(Tornesello et al., 2010)
A	MH632204	Uganda	(Zong et al., 1999)
A	MH632205	Uganda	(Zong et al., 1999)
A	MH632207	Uganda	(Zong et al., 1999)
A	MH632209	Uganda	(Zong et al., 1999)
A	MH632210	Uganda	(Zong et al., 1999)
A	MH632212	Uganda	(Zong et al., 1999)
A	MH632214	Uganda	(Zong et al., 1999)
A	MH632217	Uganda	(Zong et al., 1999)
A	MH632218	Uganda	(Zong et al., 1999)
A	MH632223	Uganda	(Zong et al., 1999)
A	MH632225	Uganda	(Zong et al., 1999)
A	MH632228	Uganda	(Zong et al., 1999)
A	MH632229	Uganda	(Zong et al., 1999)
A	MH632231	Uganda	(Zong et al., 1999)
A	MH632232	Uganda	(Zong et al., 1999)
A	MH632234	Uganda	(Zong et al., 1999)
A	MH632235	Uganda	(Zong et al., 1999)
A	MH632237	Uganda	(Zong et al., 1999)
A	MH632238	Uganda	(Zong et al., 1999)
A	MH632239	Uganda	(Zong et al., 1999)
A	MH632240	Uganda	(Zong et al., 1999)
A	AY204641	Ukrainia	(Kadyrova et al., 2003)
A	AY204657	Ukrainia	(Kadyrova et al., 2003)
A	AF133038	United States	(Nicholas et al., 1998)
A	AF133039	United States	(Nicholas et al., 1998)
A	AF151688	United States	(Meng et al., 1999)
A	AF178799	United States	(Lacoste et al., 2000a)
A	AF178807	United States	(Lacoste et al., 2000a)
A	AF278827	United States	(Meng et al., 1999)
A	AF278828	United States	(Meng et al., 1999)
A	AF278829	United States	(Meng et al., 1999)
A	AF278830	United States	(Meng et al., 1999)
A	FJ884626	United States	(Tornesello et al., 2010)
A	GU097431	United States	(Tornesello et al., 2010)
A	U86667	United States	(Lagunoff and Ganem, 1997)
B	KU950275	Argentina	(Pérez and Tous, 2017)
B	KU950276	Argentina	(Pérez and Tous, 2017)
B	KU950286	Argentina	(Pérez and Tous, 2017)
B	KU950295	Argentina	(Pérez and Tous, 2017)
B	KT215098	Brazil	(Tozetto-Mendoza et al., 2016)
B	KT215099	Brazil	(Tozetto-Mendoza et al., 2016)
B	KT215100	Brazil	(Tozetto-Mendoza et al., 2016)
B	KT215101	Brazil	(Tozetto-Mendoza et al., 2016)
B	KT215102	Brazil	(Tozetto-Mendoza et al., 2016)
B	KT215110	Brazil	(Tozetto-Mendoza et al., 2016)
B	KT215111	Brazil	(Tozetto-Mendoza et al., 2016)
B	KT215112	Brazil	(Tozetto-Mendoza et al., 2016)

B	KT215113	Brazil	(Tozetto-Mendoza et al., 2016)
B	KT215114	Brazil	(Tozetto-Mendoza et al., 2016)
B	KT215115	Brazil	(Tozetto-Mendoza et al., 2016)
B	KT215116	Brazil	(Tozetto-Mendoza et al., 2016)
B	KT215117	Brazil	(Tozetto-Mendoza et al., 2016)
B	KT215118	Brazil	(Tozetto-Mendoza et al., 2016)
B	MN318213	Brazil	(Lopes et al., 2020)
B	MN318212	Brazil	(Lopes et al., 2020)
B	MN318211	Brazil	(Lopes et al., 2020)
B	MN318214	Brazil	(Lopes et al., 2020)
B	MN318215	Brazil	(Lopes et al., 2020)
B	MN318216	Brazil	(Lopes et al., 2020)
B	AF178791	Central African Republic	(Lacoste et al., 2000a)
B	AF178792	Central African Republic	(Lacoste et al., 2000a)
B	AF178796	Central African Republic	(Lacoste et al., 2000a)
B	AF178801	Central African Republic	(Lacoste et al., 2000a)
B	AF178824	Central African Republic	(Lacoste et al., 2000a)
B	AF178783	Congo	(Lacoste et al., 2000a)
B	AY623770	Cuba	Unpublished
B	AY623775	Cuba	Unpublished
B	AY623781	Cuba	Unpublished
B	AY623784	Cuba	Unpublished
B	AY623789	Cuba	Unpublished
B	AF133040	Democratic Republic of the Congo	(Nicholas et al., 1998)
B	AF130301	England	(Cook et al., 1999)
B	AF178782	French Guiana	(Lacoste et al., 2000a)
B	AF178788	French Guiana	(Lacoste et al., 2000a)
B	AF178821	French Guiana	(Lacoste et al., 2000a)
B	AF178822	French Guiana	(Lacoste et al., 2000a)
B	AF178825	French Guiana	(Lacoste et al., 2000a)
B	AF171056	French Guiana	(Fouchard et al., 2000)
B	AF130259	Gambia	(Cook et al., 1999)
B	AF130260	Gambia	(Cook et al., 1999)
B	AF130261	Gambia	(Cook et al., 1999)
B	AF130262	Gambia	(Cook et al., 1999)
B	AF130263	Gambia	(Cook et al., 1999)
B	AF130264	Gambia	(Cook et al., 1999)
B	AF130265	Gambia	(Cook et al., 1999)
B	AF130266	Gambia	(Cook et al., 1999)
B	AF130306	Gambia	(Cook et al., 1999)
B	AF178818	Senegal	(Lacoste et al., 2000a)
B	KP997034	South Africa	(Isaacs et al., 2016)
B	KP997036	South Africa	(Isaacs et al., 2016)

B	KP997037	South Africa	(Isaacs et al., 2016)
B	KP997038	South Africa	(Isaacs et al., 2016)
B	KP997039	South Africa	(Isaacs et al., 2016)
B	KP997042	South Africa	(Isaacs et al., 2016)
B	KP997043	South Africa	(Isaacs et al., 2016)
B	KP997045	South Africa	(Isaacs et al., 2016)
B	KP997047	South Africa	(Isaacs et al., 2016)
B	KP997050	South Africa	(Isaacs et al., 2016)
B	KP997055	South Africa	(Isaacs et al., 2016)
B	KP997056	South Africa	(Isaacs et al., 2016)
B	KP997060	South Africa	(Isaacs et al., 2016)
B	KP997062	South Africa	(Isaacs et al., 2016)
B	KP997064	South Africa	(Isaacs et al., 2016)
B	KP997066	South Africa	(Isaacs et al., 2016)
B	KP997067	South Africa	(Isaacs et al., 2016)
B	KP997068	South Africa	(Isaacs et al., 2016)
B	KP997069	South Africa	(Isaacs et al., 2016)
B	KP997071	South Africa	(Isaacs et al., 2016)
B	KP997072	South Africa	(Isaacs et al., 2016)
B	KP997074	South Africa	(Isaacs et al., 2016)
B	KP997077	South Africa	(Isaacs et al., 2016)
B	KP997079	South Africa	(Isaacs et al., 2016)
B	KP997080	South Africa	(Isaacs et al., 2016)
B	KP997081	South Africa	(Isaacs et al., 2016)
B	KP997082	South Africa	(Isaacs et al., 2016)
B	KP997084	South Africa	(Isaacs et al., 2016)
B	KP997094	South Africa	(Isaacs et al., 2016)
B	KP997095	South Africa	(Isaacs et al., 2016)
B	KP997096	South Africa	(Isaacs et al., 2016)
B	KP997100	South Africa	(Isaacs et al., 2016)
B	KP997102	South Africa	(Isaacs et al., 2016)
B	KP997104	South Africa	(Isaacs et al., 2016)
B	KP997108	South Africa	(Isaacs et al., 2016)
B	KP997114	South Africa	(Isaacs et al., 2016)
B	KP997115	South Africa	(Isaacs et al., 2016)
B	KP997116	South Africa	(Isaacs et al., 2016)
B	KP997119	South Africa	(Isaacs et al., 2016)
B	KP997122	South Africa	(Isaacs et al., 2016)
B	KP997128	South Africa	(Isaacs et al., 2016)
B	KP997129	South Africa	(Isaacs et al., 2016)
B	KP997130	South Africa	(Isaacs et al., 2016)
B	MH632203	South Africa	(Zong et al., 1999)
B	AF178804	Togo	(Lacoste et al., 2000a)
B	AF130290	Uganda	(Cook et al., 1999)
B	AF130292	Uganda	(Cook et al., 1999)
B	AF130293	Uganda	(Cook et al., 1999)
B	AF151689	Uganda	(Meng et al., 1999)
B	AY042940	Uganda	(Kakoola et al., 2001)
B	AY042941	Uganda	(Kakoola et al., 2001)

B	AY042942	Uganda	(Kakoola et al., 2001)
B	AY042944	Uganda	(Kakoola et al., 2001)
B	AY042945	Uganda	(Kakoola et al., 2001)
B	AY042946	Uganda	(Kakoola et al., 2001)
B	AY042947	Uganda	(Kakoola et al., 2001)
B	AY042948	Uganda	(Kakoola et al., 2001)
B	AY042949	Uganda	(Kakoola et al., 2001)
B	FJ884618	Uganda	(Tornesello et al., 2010)
B	MH632206	Uganda	(Zong et al., 1999)
B	MH632208	Uganda	(Zong et al., 1999)
B	MH632211	Uganda	(Zong et al., 1999)
B	MH632213	Uganda	(Zong et al., 1999)
B	MH632215	Uganda	(Zong et al., 1999)
B	MH632219	Uganda	(Zong et al., 1999)
B	MH632220	Uganda	(Zong et al., 1999)
B	MH632221	Uganda	(Zong et al., 1999)
B	MH632222	Uganda	(Zong et al., 1999)
B	MH632226	Uganda	(Zong et al., 1999)
B	MH632227	Uganda	(Zong et al., 1999)
B	MH632230	Uganda	(Zong et al., 1999)
B	MH632236	Uganda	(Zong et al., 1999)
C	AF178805	Algeria	(Lacoste et al., 2000a)
C	KU950272	Argentina	(Pérez and Tous, 2017)
C	KU950274	Argentina	(Pérez and Tous, 2017)
C	KU950277	Argentina	(Pérez and Tous, 2017)
C	KU950278	Argentina	(Pérez and Tous, 2017)
C	KU950279	Argentina	(Pérez and Tous, 2017)
C	KU950280	Argentina	(Pérez and Tous, 2017)
C	KU950285	Argentina	(Pérez and Tous, 2017)
C	KU950287	Argentina	(Pérez and Tous, 2017)
C	KU950288	Argentina	(Pérez and Tous, 2017)
C	KU950290	Argentina	(Pérez and Tous, 2017)
C	KU950291	Argentina	(Pérez and Tous, 2017)
C	KU950292	Argentina	(Pérez and Tous, 2017)
C	KU950293	Argentina	(Pérez and Tous, 2017)
C	KU950294	Argentina	(Pérez and Tous, 2017)
C	KU950296	Argentina	(Pérez and Tous, 2017)
C	AF278823	Australia	(Meng et al., 1999)
C	JN800484	Australia	Unpublished
C	JN800489	Australia	Unpublished
C	KT215103	Brazil	(Tozetto-Mendoza et al., 2016)
C	KT215104	Brazil	(Tozetto-Mendoza et al., 2016)
C	KT215105	Brazil	(Tozetto-Mendoza et al., 2016)
C	KT215119	Brazil	(Tozetto-Mendoza et al., 2016)
C	KT215120	Brazil	(Tozetto-Mendoza et al., 2016)
C	KT215121	Brazil	(Tozetto-Mendoza et al., 2016)
C	KT215122	Brazil	(Tozetto-Mendoza et al., 2016)
C	KT215124	Brazil	(Tozetto-Mendoza et al., 2016)
C	MN318222	Brazil	(Lopes et al., 2020)

C	MN318223	Brazil	(Lopes et al., 2020)
C	MN318224	Brazil	(Lopes et al., 2020)
C	MN318225	Brazil	(Lopes et al., 2020)
C	MN318226	Brazil	(Lopes et al., 2020)
C	MN318220	Brazil	(Lopes et al., 2020)
C	MN318219	Brazil	(Lopes et al., 2020)
C	MN318221	Brazil	(Lopes et al., 2020)
C	MN318218	Brazil	(Lopes et al., 2020)
C	MN318227	Brazil	(Lopes et al., 2020)
C	MN318217	Brazil	(Lopes et al., 2020)
C	AF178780	Central African Republic	(Lacoste et al., 2000a)
C	AF178789	Central African Republic	(Lacoste et al., 2000a)
C	AY623777	Cuba	Unpublished
C	AF130298	England	(Cook et al., 1999)
C	AF130300	England	(Cook et al., 1999)
C	AF130302	England	(Cook et al., 1999)
C	AF130304	England	(Cook et al., 1999)
C	AF042370	France	(Morand et al., 1999)
C	AF178773	France	(Lacoste et al., 2000a)
C	AF178774	France	(Lacoste et al., 2000a)
C	AF178777	France	(Lacoste et al., 2000a)
C	AF178781	France	(Lacoste et al., 2000a)
C	AF178787	France	(Lacoste et al., 2000a)
C	AF178793	France	(Lacoste et al., 2000a)
C	AF178795	France	(Lacoste et al., 2000a)
C	AF178800	France	(Lacoste et al., 2000a)
C	AF178803	France	(Lacoste et al., 2000a)
C	AF178806	France	(Lacoste et al., 2000a)
C	AF178808	France	(Lacoste et al., 2000a)
C	AF178828	France	(Lacoste et al., 2000a)
C	AF178776	French Guiana	(Lacoste et al., 2000a)
C	AF130267	Greece	(Cook et al., 1999)
C	AF130268	Greece	(Cook et al., 1999)
C	GU097417	Greece	(Tornesello et al., 2010)
C	GU097423	Greece	(Tornesello et al., 2010)
C	AF130273	Iceland	(Cook et al., 1999)
C	JN242286	Iran	(Jalilvand et al., 2012)
C	JN242287	Iran	(Jalilvand et al., 2012)
C	JN242288	Iran	(Jalilvand et al., 2012)
C	JN242289	Iran	(Jalilvand et al., 2012)
C	AF130270	Italy	(Cook et al., 1999)
C	AF130271	Italy	(Cook et al., 1999)
C	AF130274	Italy	(Cook et al., 1999)
C	AF130286	Italy	(Cook et al., 1999)
C	FJ884614	Italy	(Tornesello et al., 2010)
C	GU097425	Italy	(Tornesello et al., 2010)
C	HQ391937	Italy	(Mancuso et al., 2011)

C	HQ391939	Italy	(Mancuso et al., 2011)
C	HQ391942	Italy	(Mancuso et al., 2011)
C	HQ391943	Italy	(Mancuso et al., 2011)
C	HQ391945	Italy	(Mancuso et al., 2011)
C	HQ391946	Italy	(Mancuso et al., 2011)
C	AF278840	Japan	(Meng et al., 2001)
C	AF278841	Japan	(Meng et al., 2001)
C	AF278843	Japan	(Meng et al., 2001)
C	AF278847	Japan	(Meng et al., 2001)
C	AF278848	Japan	(Meng et al., 2001)
C	AF278849	Japan	(Meng et al., 2001)
C	AF278850	Japan	(Meng et al., 2001)
C	GU097418	Kenya	(Tornesello et al., 2010)
C	DQ394034	Marocco	(Duprez et al., 2006b)
C	DQ394035	Marocco	(Duprez et al., 2006b)
C	DQ394036	Marocco	(Duprez et al., 2006b)
C	DQ394039	Marocco	(Duprez et al., 2006b)
C	DQ394040	Marocco	(Duprez et al., 2006b)
C	DQ394041	Marocco	(Duprez et al., 2006b)
C	DQ394042	Marocco	(Duprez et al., 2006b)
C	DQ394043	Marocco	(Duprez et al., 2006b)
C	DQ394044	Marocco	(Duprez et al., 2006b)
C	DQ394045	Marocco	(Duprez et al., 2006b)
C	DQ394046	Marocco	(Duprez et al., 2006b)
C	DQ394047	Marocco	(Duprez et al., 2006b)
C	DQ394048	Marocco	(Duprez et al., 2006b)
C	DQ394049	Marocco	(Duprez et al., 2006b)
C	DQ394050	Marocco	(Duprez et al., 2006b)
C	DQ394051	Marocco	(Duprez et al., 2006b)
C	DQ394052	Marocco	(Duprez et al., 2006b)
C	DQ394053	Marocco	(Duprez et al., 2006b)
C	DQ394054	Marocco	(Duprez et al., 2006b)
C	DQ394055	Marocco	(Duprez et al., 2006b)
C	DQ394056	Marocco	(Duprez et al., 2006b)
C	DQ394058	Marocco	(Duprez et al., 2006b)
C	DQ394059	Marocco	(Duprez et al., 2006b)
C	DQ394060	Marocco	(Duprez et al., 2006b)
C	DQ394061	Marocco	(Duprez et al., 2006b)
C	DQ394062	Marocco	(Duprez et al., 2006b)
C	DQ394063	Marocco	(Duprez et al., 2006b)
C	DQ394065	Marocco	(Duprez et al., 2006b)
C	DQ394066	Marocco	(Duprez et al., 2006b)
C	DQ394067	Marocco	(Duprez et al., 2006b)
C	AF201851	Russia	(Lacoste et al., 2000b)
C	AY204642	Russia	(Kadyrova et al., 2003)
C	AY204649	Russia	(Kadyrova et al., 2003)
C	AY204661	Russia	(Kadyrova et al., 2003)
C	AY204666	Russia	(Kadyrova et al., 2003)
C	AY204669	Russia	(Kadyrova et al., 2003)

C	AM423130	Saudi Arabia	(Al-Otaibi et al., 2007)
C	AM423135	Saudi Arabia	(Al-Otaibi et al., 2007)
C	AM423136	Saudi Arabia	(Al-Otaibi et al., 2007)
C	AF178820	Senegal	(Lacoste et al., 2000a)
C	AY766082	Tunisia	Unpublished
C	AY766083	Tunisia	Unpublished
C	AY042954	Uganda	(Kakoola et al., 2001)
C	GU097424	Uganda	(Tornesello et al., 2010)
C	MH632224	Uganda	(Zong et al., 1999)
C	MH632233	Uganda	(Zong et al., 1999)
C	AY204659	Ukrainia	(Kadyrova et al., 2003)
C	AF133041	United States	(Nicholas et al., 1998)
C	AF133042	United States	(Nicholas et al., 1998)
C	AF151686	United States	(Meng et al., 1999)
C	AF170531	United States	(Samaniego et al., 2001)
C	AF278826	United States	(Meng et al., 1999)
C	GU097422	United States	(Tornesello et al., 2010)
D	AF151687	Australia	(Meng et al., 1999)
D	AF278844	Japan	(Meng et al., 2001)
D	AF278845	Japan	(Meng et al., 2001)
D	AF278846	Japan	(Meng et al., 2001)
D	AF133044	Pacific island	(Zong et al., 1999)
D	AF133043	Taiwan	(Zong et al., 1999)
D	DQ386448	Wallis Island	(Duprez et al., 2006a)
E	AF220292	Brazil	(Biggar et al., 2000)
E	AF220293	Brazil	(Biggar et al., 2000)
E	AY329026	Ecuador	(Whitby et al., 2004)
E	AY329027	Ecuador	(Whitby et al., 2004)
E	AY329028	Ecuador	(Whitby et al., 2004)
F	KT215106	Brazil	(Tozetto-Mendoza et al., 2016)
F	AF178810	France	(Lacoste et al., 2000a)
F	MK876732	France	(Jary et al., 2020)
F	MK876734	France	(Jary et al., 2020)
F	MK876735	France	(Jary et al., 2020)
F	MK876736	France	(Jary et al., 2020)
F	FJ884616	Kenya	(Tornesello et al., 2010)
F	KP997076	South Africa	(Isaacs et al., 2016)
F	KP997134	South Africa	(Isaacs et al., 2016)
F	MH632216	Uganda	(Kajumbula, et al., 2006)

Note: *Unpublished or in preparation: Sequences unpublished on PubMed. Information collected from GenBank.

References

1. Al-Otaibi, L.M., Ngui, S.L., Scully, C.M., Porter, S.R., Teo, C.G., 2007. Salivary human herpesvirus 8 shedding in renal allograft recipients with Kaposi's sarcoma. J Med Virol 79(9), 1357-1365.
2. Biggar, R.J., Whitby, D., Marshall, V., Linhares, A.C., Black, F., 2000. Human herpesvirus 8 in Brazilian Amerindians: a hyperendemic population with a new subtype. J Infect Dis 181(5), 1562-1568.
3. Cook, P.M., Whitby, D., Calabro, M.L., Luppi, M., Kakoola, D.N., Hjalgrim, H., Ariyoshi, K., Ensoli, B., Davison, A.J., Schulz, T.F., 1999. Variability and evolution of Kaposi's sarcoma-associated herpesvirus in Europe and Africa. International Collaborative Group. AIDS 13(10), 1165-1176.
4. Lopes, A.O, Spitz, N., Martinelli, K.G., de Paula, A.V., de Castro Conde Toscano, A.L., Braz-Silva, P.H., Dos Santos Barbosa Netto, J., Tozetto-Mendoza, T.R., de Paula, V.S., 2020. Introduction of human gammaherpesvirus 8 genotypes A, B, and C into Brazil from multiple geographic regions. Virus Res 276, 197828.
5. Duprez, R., Cassar, O., Hbid, O., Rougier, Y., Morisse, L., Bassot, S., Huerre, M., Gessain, A., 2006a. Cutaneous disseminated endemic Kaposi's sarcoma in a Polynesian man infected with a new divergent human herpesvirus 8 subtype D. J Clin Virol 37(3), 222-226.
6. Duprez, R., Hbid, O., Afonso, P., Quach, H., Belloul, L., Fajali, N., Ismaili, N., Benomar, H., Hassane Tahri, E., Huerre, M., Quintana-Murci, L., Gessain, A., 2006b. Molecular epidemiology of the HHV-8 K1 gene from Moroccan patients with Kaposi's sarcoma. Virology 353(1), 121-132.
7. Fouchard, N., Lacoste, V., Couppie, P., Develoux, M., Mauclere, P., Michel, P., Herve, V., Pradinaud, R., Bestetti, G., Huerre, M., Tekaia, F., de Thé, G., Gessain, A., 2000. Detection and genetic polymorphism of human herpes virus type 8 in endemic or epidemic Kaposi's sarcoma from West and Central Africa, and South America. Int J Cancer 85(2), 166-170.
8. Isaacs, T., Abera, A.B., Muloiwa, R., Katz, A.A., Todd, G., 2016. Genetic diversity of HHV8 subtypes in South Africa: A5 subtype is associated with extensive disease in AIDS-KS. J Med Virol 88(2), 292-303.
9. Jalilvand, S., Tornesello, M.L., Buonaguro, F.M., Buonaguro, L., Naraghi, Z.S., Shoja, Z., Ziaee, A.A., Hamkar, R., Shahmahmoodi, S., Nategh, R., Mokhtari-Azad, T., 2012. Molecular epidemiology of human herpesvirus 8 variants in Kaposi's sarcoma from Iranian patients. Virus Res 163(2), 644-649.
10. Kadyrova, E., Lacoste, V., Duprez, R., Pozharissky, K., Molochkov, V., Huerre, M., Gurtsevitch, V., Gessain, A., 2003. Molecular epidemiology of Kaposi's sarcoma-associated herpesvirus/human herpesvirus 8 strains from Russian patients with classic, posttransplant, and AIDS-associated Kaposi's sarcoma. J Med Virol 71(4), 548-556.
11. Kajumbula, H., Wallace, R.G., Zong, J.C., Hokello, J., Sussman, N., Simms, S., Rockwell, R.F., Pozos, R., Hayward, G.S., Boto, W. 2006. Ugandan Kaposi's Sarcoma-Associated Herpesvirus Phylogeny: Evidence for Cross-Ethnic Transmission of Viral Subtypes. Intervirology 49,133-143.
12. Kakoola, D.N., Sheldon, J., Byabazaire, N., Bowden, R.J., Katongole-Mbidde, E., Schulz, T.F., Davison, A.J., 2001. Recombination in human herpesvirus-8 strains from Uganda and evolution of the K15 gene. J Gen Virol 82(Pt 10), 2393-2404.
13. Lacoste, V., Judde, J.G., Brière, J., Tulliez, M., Garin, B., Kassa-Kelembho, E., Morvan, J., Couppié, P., Clyti, E., Forteza Vila, J., Rio, B., Delmer, A., Mauclère, P.,

Gessain, A., 2000a. Molecular epidemiology of human herpesvirus 8 in africa: both B and A5 K1 genotypes, as well as the M and P genotypes of K14.1/K15 loci, are frequent and widespread. Virology 278(1), 60-74.

14. Lacoste, V., Kadyrova, E., Chistiakova, I., Gurtsevitch, V., Judde, J.G., Gessain, A., 2000b. Molecular characterization of Kaposi's sarcoma-associated herpesvirus/human herpesvirus-8 strains from Russia. J Gen Virol 81(Pt 5), 1217-1222.
15. Lagunoff, M., Ganem, D., 1997. The structure and coding organization of the genomic termini of Kaposi's sarcoma-associated herpesvirus. Virology 236(1), 147-154.
16. Mancuso, R., Brambilla, L., Agostini, S., Biffi, R., Hernis, A., Guerini, F.R., Agliardi, C., Tourlaki, A., Bellinvia, M., Clerici, M., 2011. Intrafamiliar transmission of Kaposi's sarcoma-associated herpesvirus and seronegative infection in family members of classic Kaposi's sarcoma patients. J Gen Virol 92(Pt 4), 744-751.
17. Meng, Y.X., Sata, T., Stamey, F.R., Voevodin, A., Katano, H., Koizumi, H., Deleon, M., De Cristofano, M.A., Galimberti, R., Pellett, P.E., 2001. Molecular characterization of strains of Human herpesvirus 8 from Japan, Argentina and Kuwait. J Gen Virol 82(Pt 3), 499-506.
18. Meng, Y.X., Spira, T.J., Bhat, G.J., Birch, C.J., Druce, J.D., Edlin, B.R., Edwards, R., Gunthel, C., Newton, R., Stamey, F.R., Wood, C., Pellett, P.E., 1999. Individuals from North America, Australasia, and Africa are infected with four different genotypes of human herpesvirus 8. Virology 261(1), 106-119.
19. Morand, P., Buisson, M., Collandre, H., Chanzy, B., Genoulaz, O., Bourgeat, M.J., Pinel, N., Leclercq, P., Leroux, D., Marechal, V., Fritsch, L., Ruigrok, R., Seigneurin, J.M., 1999. Human herpesvirus 8 and Epstein Barr-virus in a cutaneous B-cell lymphoma and a malignant cell line established from the blood of an AIDS patient. Leuk Lymphoma 35(3-4), 379-387.
20. Nicholas, J., Zong, J.C., Alcendor, D.J., Ciufo, D.M., Poole, L.J., Sarisky, R.T., Chiou, C.J., Zhang, X., Wan, X., Guo, H.G., Reitz, M.S., Hayward, G.S., 1998. Novel organizational features, captured cellular genes, and strain variability within the genome of KSHV/HHV8. J Natl Cancer Inst Monogr(23), 79-88.
21. Pérez, C.L., Tous, M.I., 2017. Diversity of human herpesvirus 8 genotypes in patients with AIDS and non-AIDS associated Kaposi's sarcoma, Castleman's disease and primary effusion lymphoma in Argentina. J Med Virol 89(11), 2020-2028.
22. Samaniego, F., Pati, S., Karp, J.E., Prakash, O., Bose, D., 2001. Human herpesvirus 8 K1-associated nuclear factor-kappa B-dependent promoter activity: role in Kaposi's sarcoma inflammation? J Natl Cancer Inst Monogr(28), 15-23.
23. Shirokov, D., Kadyrova, E., Anokhina, M., Kondratyeva, T., Gourtsevich, V., Tupitsyn, N., 2007. A case of HHV-8-associated HIV-negative primary effusion lymphoma in Moscow. J Med Virol 79(3), 270-277.
24. Speicher, D.J., Sehu, M.M., Johnson, N.W., Shaw, D.R., 2013. Successful treatment of an HIV-positive patient with unmasking Kaposi's sarcoma immune reconstitution inflammatory syndrome. J Clin Virol 57(3), 282-285.
25. Tornesello, M.L., Biryahwaho, B., Downing, R., Hatzakis, A., Alessi, E., Cusini, M., Ruocco, V., Katongole-Mbidde, E., Loquercio, G., Buonaguro, L., Buonaguro, F.M., 2010. Human herpesvirus type 8 variants circulating in Europe, Africa and North America in classic, endemic and epidemic Kaposi's sarcoma lesions during pre-AIDS and AIDS era. Virology 398(2), 280-289.
26. Tozetto-Mendoza, T.R., Ibrahim, K.Y., Tateno, A.F., Oliveira, C.M., Sumita, L.M., Sanchez, M.C., Luna, E.J., Pierrotti, L.C., Drexler, J.F., Braz-Silva, P.H., Pannuti,

C.S., Romano, C.M., 2016. Genotypic distribution of HHV-8 in AIDS individuals without and with Kaposi sarcoma: Is genotype B associated with better prognosis of AIDS-KS? Medicine (Baltimore) 95(48), e5291.
27. Treurnicht, F.K., Engelbrecht, S., Taylor, M.B., Schneider, J.W., van Rensburg, E.J., 2002. HHV-8 subtypes in South Africa: identification of a case suggesting a novel B variant. J Med Virol 66(2), 235-240.
28. Whitby, D., Marshall, V.A., Bagni, R.K., Wang, C.D., Gamache, C.J., Guzman, J.R., Kron, M., Ebbesen, P., Biggar, R.J., 2004. Genotypic characterization of Kaposi's sarcoma-associated herpesvirus in asymptomatic infected subjects from isolated populations. J Gen Virol 85(Pt 1), 155-163.
29. Zong, J.C., Ciufo, D.M., Alcendor, D.J., Wan, X., Nicholas, J., Browning, P.J., Rady, P.L., Tyring, S.K., Orenstein, J.M., Rabkin, C.S., Su, I.J., Powell, K.F., Croxson, M., Foreman, K.E., Nickoloff, B.J., Alkan, S., Hayward, G.S., 1999. High-level variability in the ORF-K1 membrane protein gene at the left end of the Kaposi's sarcoma-associated herpesvirus genome defines four major virus subtypes and multiple variants or clades in different human populations. J Virol 73(5), 4156-4170.
30. Jary, A., Leducq, V., Desire, N., Petit, H., Palich, R., Joly, V., Canestri, A., Gothland, A., Lambert-Niclot, S., Surgers, L., Amiel, C., Descamps, D., Spano, J.P., Katlama, C., Calvez, V., Marcelin, A.G., 2020. New Kaposi’s sarcoma-associated herpesvirus variant in men who have sex with men associated with severe pathologies. J Infect Dis 222, 1320-1328.
