# Supplementary figures and images for: Update of the global distribution of human gammaherpesvirus 8 genotypes
Source: Sci Rep. 2021 Apr 7;11:7640. doi: 10.1038/s41598-021-87038-9 (PMC8026617; doi:10.1038/s41598-021-87038-9)

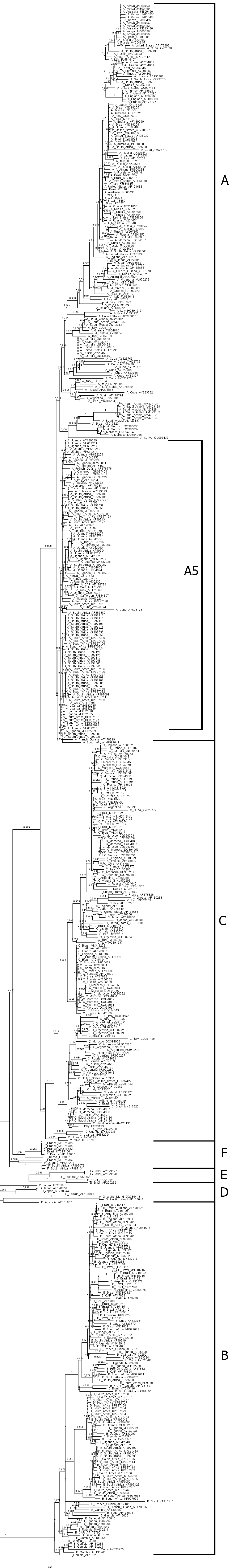

Supplement: Supplementary file 2 — Supplementary Information 2. [file 41598_2021_87038_MOESM2_ESM.png]
